# Life-threatening diffuse alveolar hemorrhage associated with *Rhinovirus infection* and the potential role of corticosteroids in immunocompetent patients: Case reports

**DOI:** 10.1097/MD.0000000000051157

**Published:** 2026-10-16

**Authors:** Chang-Seok Yoon, Joon-Young Yoon, Bo-Gun Kho, Tae-Ok Kim, Yong-Soo Kwon, Yu-Il Kim, Sung-Chul Lim, Hong-Joon Shin

**Affiliations:** a Department of Internal Medicine, Chonnam National University Hospital, Gwangju, Republic of Korea; b Chonnam National University Medical School, Gwangju, Republic of Korea.

**Keywords:** case report, corticosteroid, diffuse alveolar hemorrhage, rhinovirus

## Abstract

**Rationale:**

Diffuse alveolar hemorrhage (DAH) is a rare but potentially fatal condition characterized by bleeding into alveolar spaces, most often associated with autoimmune disease or severe infection. Rhinovirus is a common cause of upper respiratory tract infection but is rarely associated with DAH in immunocompetent individuals. We report 2 cases of life-threatening DAH associated with rhinovirus infection, both presenting with severe hypoxemia and showing marked improvement following corticosteroid therapy.

**Patient concerns:**

Case 1: An 85-year-old woman presented with massive hemoptysis, dyspnea, and hypoxemia. Chest computed tomography (CT) revealed diffuse ground-glass opacities (GGOs) and patchy consolidations. Case 2: A 70-year-old woman developed worsening dyspnea and hemoptysis despite antibiotics. She was hypoxemic, and chest CT showed bilateral GGOs and consolidations.

**Diagnoses:**

Bronchoalveolar lavage fluid demonstrated progressively hemorrhagic aliquots consistent with DAH in both cases. Multiplex viral polymerase chain reaction identified rhinovirus as the sole pathogen in both cases, and autoimmune serologic tests were negative.

**Interventions:**

Both patients received high-flow oxygen therapy, empirical antibiotics, and systemic corticosteroids (Case 1: intravenous methylprednisolone 1 mg/kg/day; Case 2: intravenous methylprednisolone 500 mg/day for 3 days, followed by tapering).

**Outcomes:**

After corticosteroid therapy, both patients showed rapid clinical and radiographic improvement, with resolution of hypoxemia and discharge within 10 days.

**Lessons:**

These cases suggest a potential association between rhinovirus infection and life-threatening DAH in immunocompetent patients. Both patients showed marked clinical improvement after corticosteroid therapy, suggesting a potential therapeutic role for systemic corticosteroids. Thus, clinicians should consider rhinovirus infection as a possible infectious trigger of DAH, and systemic corticosteroids may be considered in selected severe cases.

## 1. Introduction

Diffuse alveolar hemorrhage (DAH) is characterized by the leakage of blood from pulmonary capillaries into the alveolar spaces, typically resulting from damage to the alveolar-capillary membrane or abnormalities in coagulation mechanisms.^[1,2]^ This condition can progress to acute respiratory distress syndrome and is associated with a high in-hospital mortality rate of 20 to 100%, rendering it potentially life-threatening.^[1,3]^ DAH most commonly occurs in systemic autoimmune diseases, such as anti-neutrophil cytoplasmic antibody (ANCA)-associated vasculitis,^[4]^ but can also result from respiratory infections. Reported pathogens include *Streptococcus pneumoniae*, *Haemophilus influenzae*, *Klebsiella pneumoniae*, *Pseudomonas aeruginosa*, SARS-CoV-2, and influenza virus,^[5]^ all of which primarily affect the lower respiratory tract.

Here, we describe 2 cases of life-threatening DAH associated with rhinovirus infection in immunocompetent patients and highlight the potential role of corticosteroid therapy in improving clinical outcomes.

## 2. Case report

### 2.1. Case 1

An 85-year-old woman presented to the emergency department with hemoptysis. She was a never-smoker with a history of hypertension and was not taking antiplatelet or anticoagulant agents or any other medications known to increase bleeding risk. She presented with approximately 200 mL of hemoptysis, consistent with massive hemoptysis, along with cough and dyspnea.

On physical examination, she was tachycardic at 120 beats/minutes, with a blood pressure of 100/65 mm Hg, body temperature of 36.4°C, and a respiratory rate of 20 breaths/minutes. A chest examination showed coarse crackles in both lungs.

Laboratory tests showed a white blood cell count of 10,300/µL, hemoglobin 15.0 g/dL, platelet count 141,000/µL, and C-reactive protein 0.1 mg/dL. Coagulation studies revealed a prothrombin time of 10.7 seconds, an activated partial thromboplastin time of 25.5 seconds, and a fibrinogen level of 313.4 mg/dL. D-dimer (1.11 µg/mL) and fibrin degradation products (5.7 µg/mL) were mildly elevated. Serum pro-brain natriuretic peptide (BNP) level was 425 pg/mL. Chest radiography and computed tomography (CT) showed diffuse ground-glass opacities (GGOs) and patchy consolidations in both lungs without structural abnormalities (Figs. 1 and 2). Consequently, cardiogenic pulmonary edema was considered unlikely based on the CT findings, serum pro-BNP, and the absence of volume overload.

**Figure 1.**
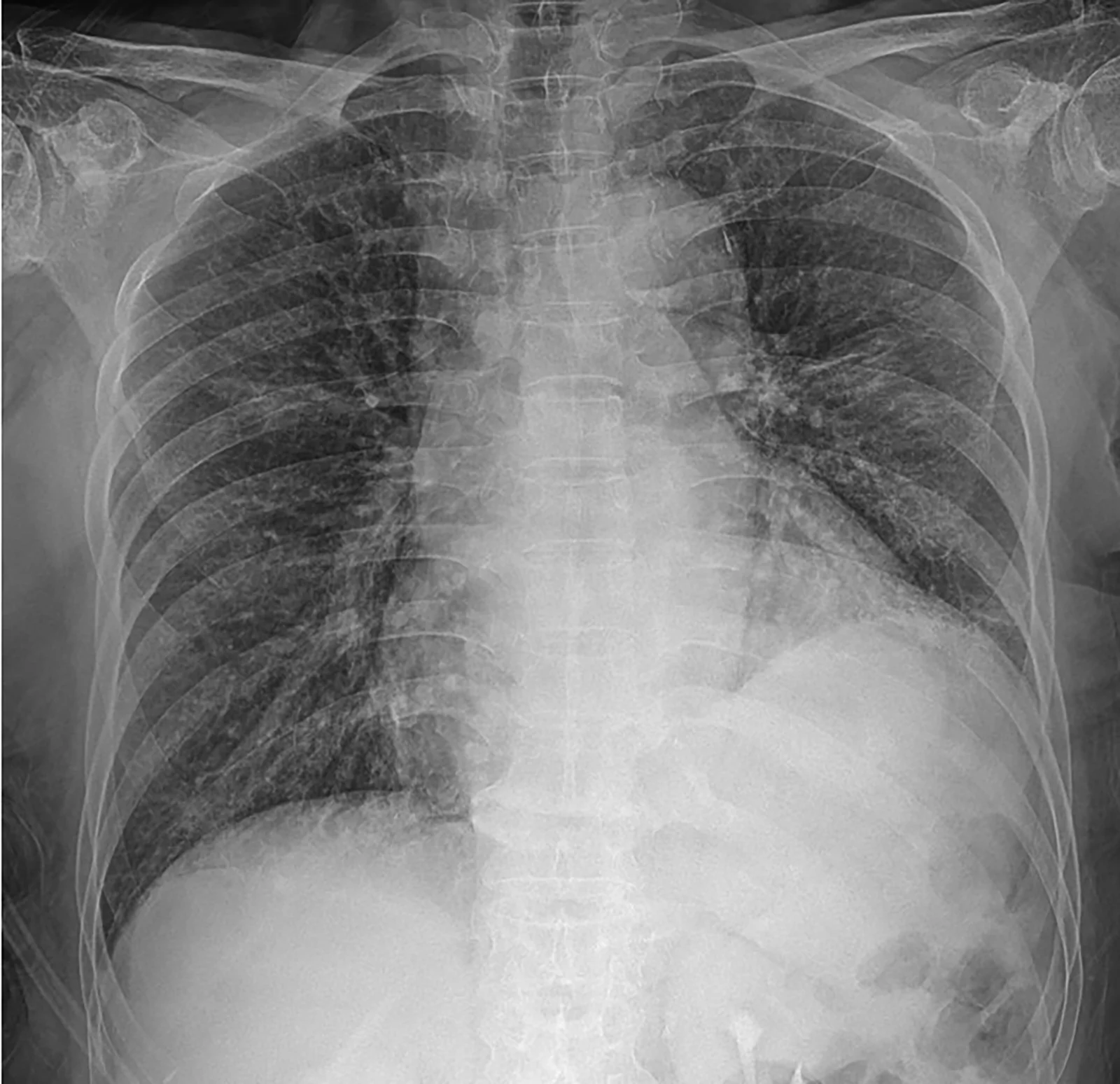
Initial chest X-ray of Case 1, showing bilateral infiltrates.

**Figure 2.**
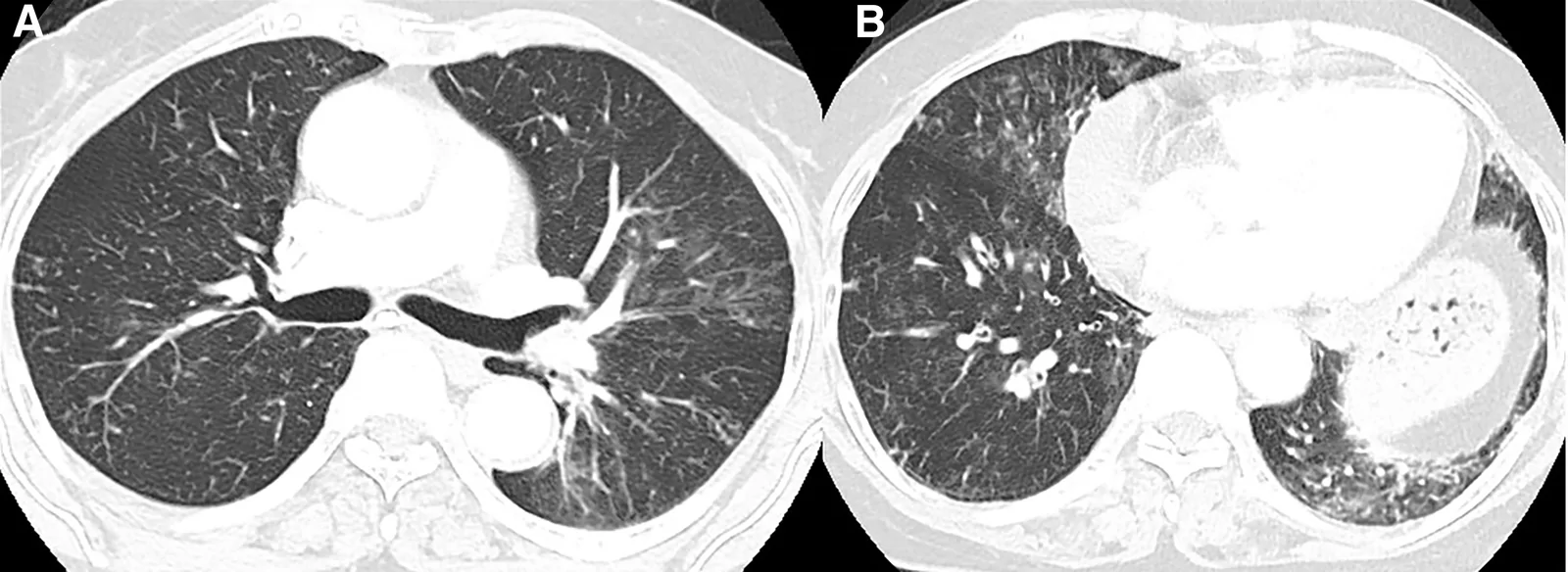
Initial chest computed tomography of Case 1, showing bilateral ground-glass opacities and consolidations in both lungs (A, B).

She received oxygen via high-flow nasal cannula therapy for severe hypoxemia. On arterial blood gas analysis, the arterial partial pressure of oxygen to fraction of inspired oxygen ratio (PaO_2_/FiO_2_ ratio) was 105 mm Hg. Given her clinical severity, empirical piperacillin/tazobactam and levofloxacin were initiated, immediately followed by bronchoscopy with bronchoalveolar lavage (BAL) to determine the underlying etiology.

The BAL fluid appeared progressively hemorrhagic, consistent with alveolar hemorrhage (Fig. 3). Quantitative BAL analysis showed a red blood cell count of 423,600 cells/µL and a total nucleated cell count of 570 cells/µL. Given the massive hemoptysis, severe hypoxemia, and bronchoscopic findings consistent with DAH, intravenous methylprednisolone was immediately initiated at a conventional dose of 1 mg/kg/day based on the treating physician’s clinical judgment, with subsequent tapering guided by clinical improvement. After initiating corticosteroid therapy, her clinical course improved dramatically, accompanied by improvement on chest radiograph (Fig. 4), allowing for successful oxygen weaning 3 days after starting corticosteroids. Microbiological evaluations showed no evidence of bacterial infection (based on negative BAL fluid Gram staining and culture), fungal infection (based on negative BAL fluid fungal culture and galactomannan assay and serum β-D-glucan), or mycobacterial infection (based on negative BAL fluid acid-fast bacillus staining, culture, and polymerase chain reaction [PCR]). In contrast, multiplex respiratory viral PCR of the BAL fluid detected rhinovirus. Serologic tests for autoimmune markers, including anti-nuclear antibody and ANCA, were negative. Her hemoglobin level decreased from 15.0 g/dL on admission to a nadir of 11.3 g/dL on day 4 post-hospitalization. Despite this decline, no blood transfusion was required. She was discharged on hospital day 10 on maintenance oral prednisolone 20 mg/day. At the 2-week follow-up, the prednisolone dose was reduced to 15 mg/day, followed by 10 mg/day for 1 month and 5 mg/day for 1 month. Afterward, corticosteroid therapy was discontinued. No recurrent hemoptysis occurred after corticosteroid discontinuation. Figure 5 provides a timeline of the patient’s clinical course.

**Figure 3.**
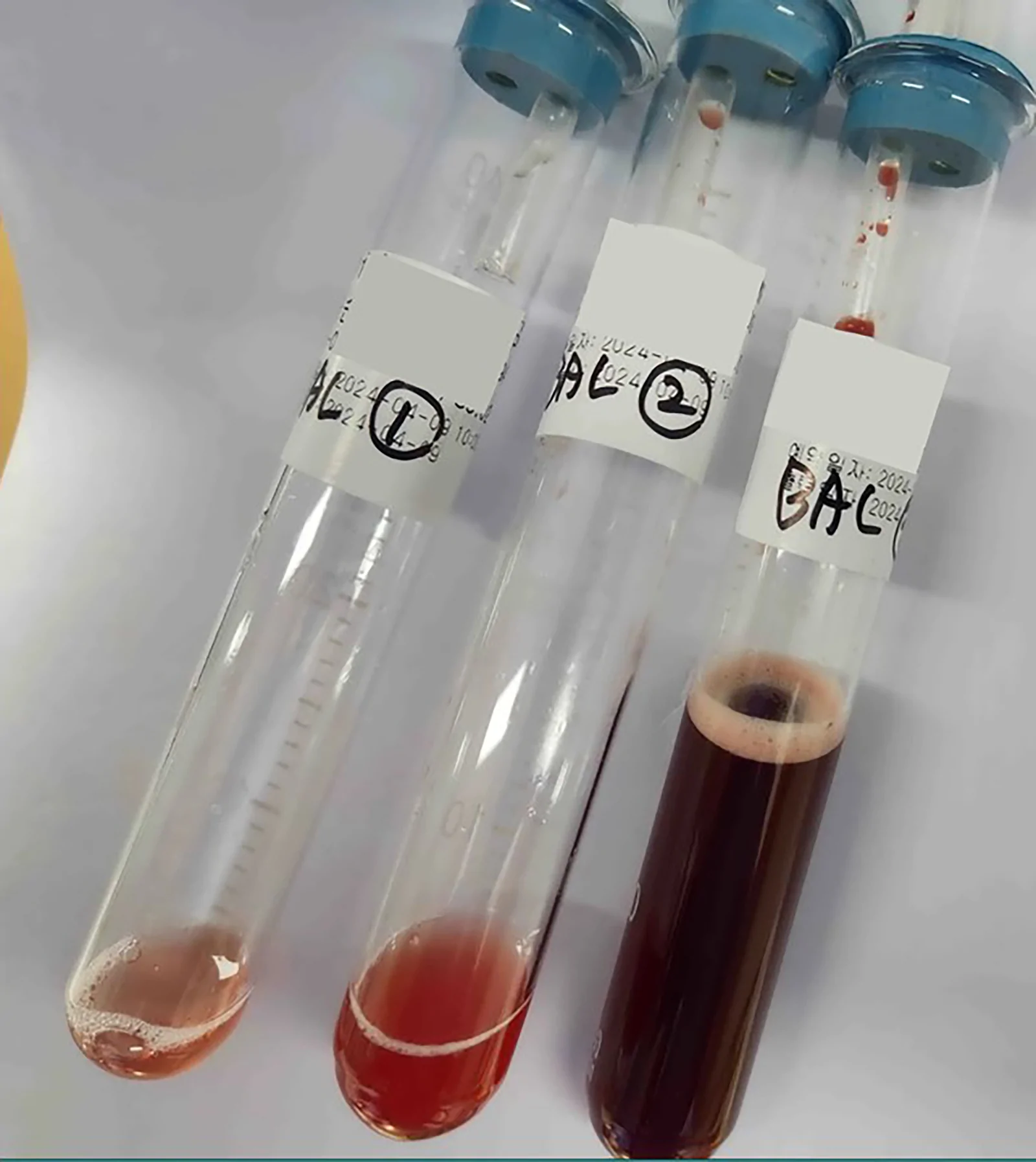
Bronchoalveolar lavage fluid from Case 1, showing a progressively hemorrhagic appearance consistent with alveolar hemorrhage.

**Figure 4.**
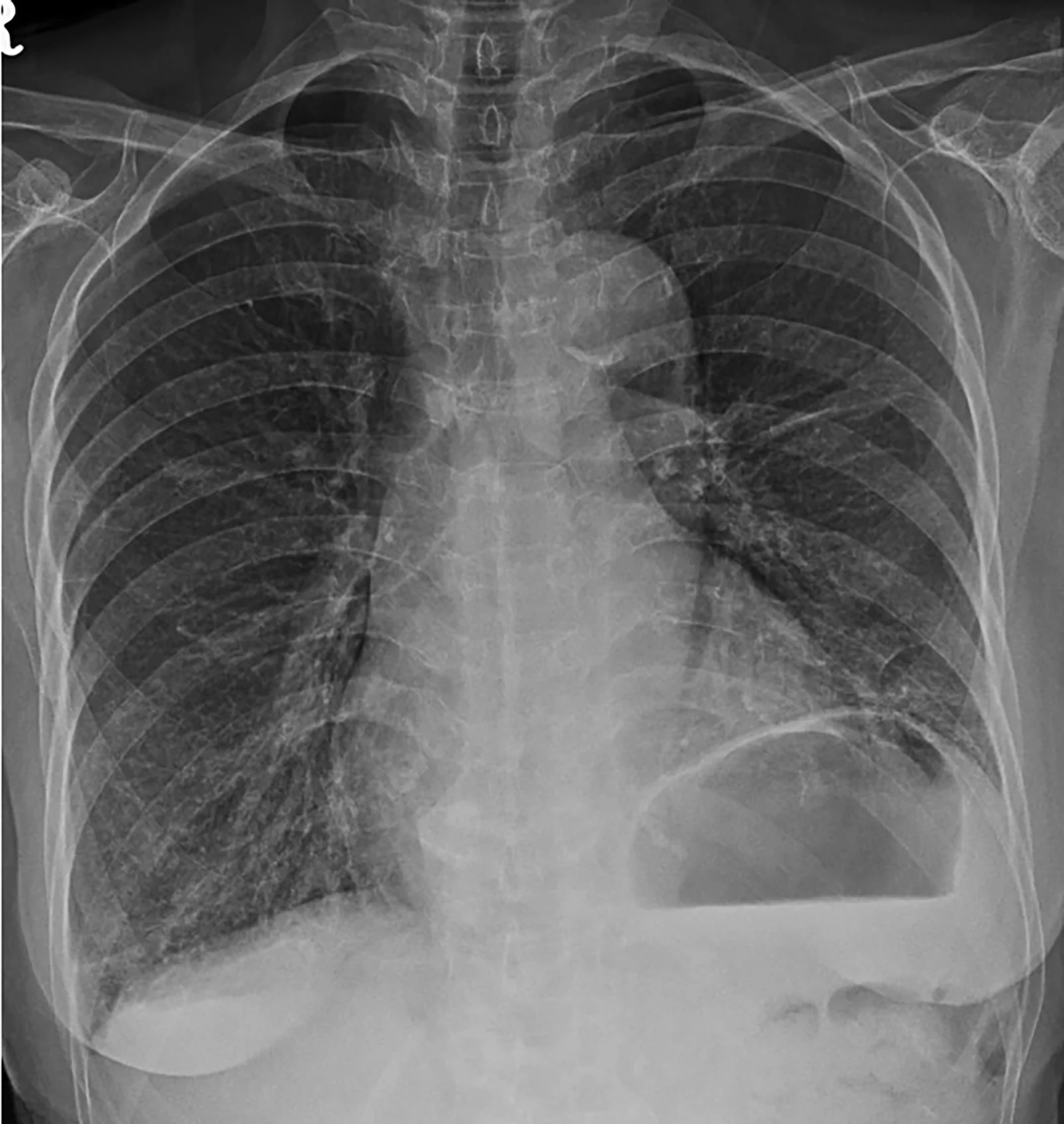
Chest radiograph of Case 1 on day 3 of corticosteroid therapy, demonstrating improvement of bilateral infiltrates with clinical recovery.

**Figure 5.**
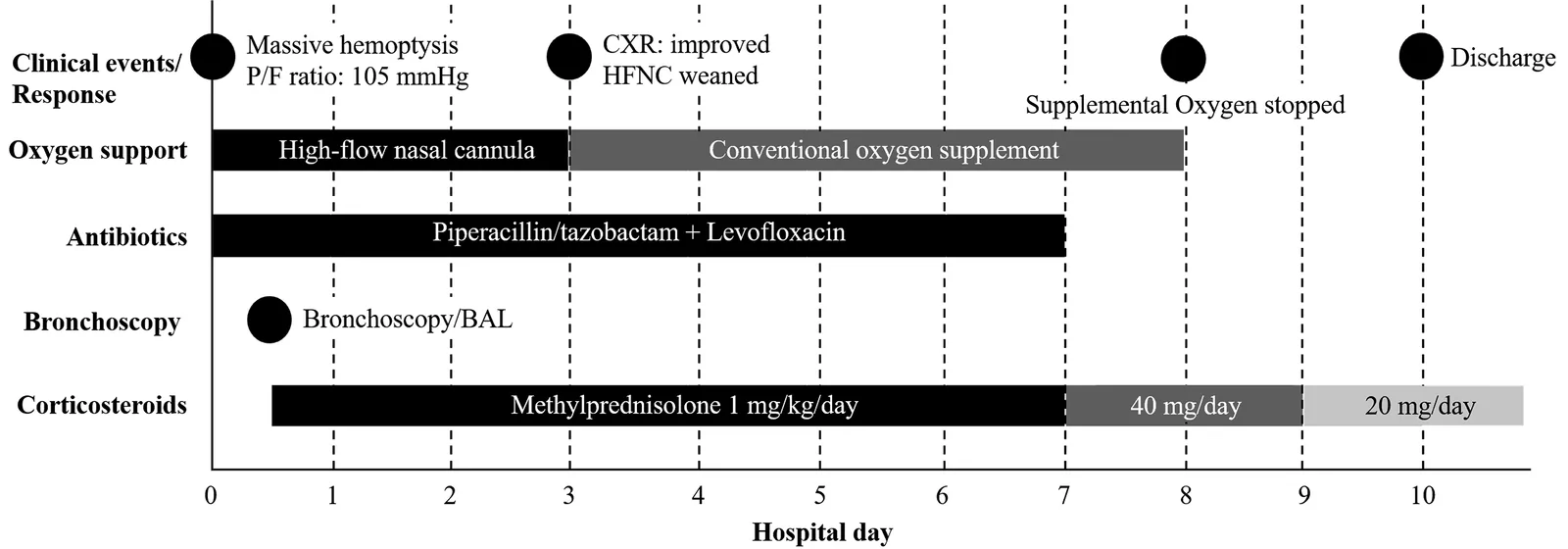
Timeline of the patient’s clinical course. BAL = bronchoalveolar lavage, CXR = chest X-ray, HFNC = high-flow nasal cannula, P/F ratio = the arterial partial pressure of oxygen to fraction of inspired oxygen ratio.

### 2.2. Case 2

A 70-year-old woman presented to the emergency department with dyspnea and hemoptysis. She was a never-smoker with a history of hypertension, dyslipidemia, and asthma, as well as a previous superficial parotidectomy for a benign parotid gland tumor. She was not taking antiplatelet or anticoagulant agents. There was no evidence of recent medication changes, nonsteroidal anti-inflammatory drug use, or inhalation exposure associated with hemoptysis. Five days before admission, she had been hospitalized at a local clinic, where she received antibiotics for fever, chills, and myalgia. Despite treatment, her symptoms worsened, and on the day of transfer, she developed hemoptysis and progressive dyspnea. At presentation, the volume of hemoptysis was approximately 150 mL.

Initial vital signs were: blood pressure 130/70 mm Hg; body temperature 38.0°C; heart rate 84 beats/minutes; and respiratory rate 26 breaths/minutes. Physical examination revealed crackles in both lung fields.

Laboratory findings included a white blood cell count of 11,700/µL, hemoglobin 11.8 g/dL, platelet count 283,000/µL, and C-reactive protein 32.24 mg/dL. Coagulation tests showed a prothrombin time of 13.4 seconds, partial thromboplastin time 28.2 seconds, fibrin degradation products 40.4 µg/mL, fibrinogen 900 mg/dL, and D-dimer 18.14 µg/mL. Serum pro-BNP level was 38 pg/mL. Although D-dimer and fibrin degradation products were elevated, the preserved platelet count, fibrinogen level, and prothrombin time/partial thromboplastin time did not support the diagnosis of overt disseminated intravascular coagulation. Chest radiography and CT demonstrated diffuse bilateral GGOs and consolidations (Figs. 6 and 7). Consequently, cardiogenic pulmonary edema was considered unlikely based on radiological findings, serum pro-BNP, and the absence of volume overload.

**Figure 6.**
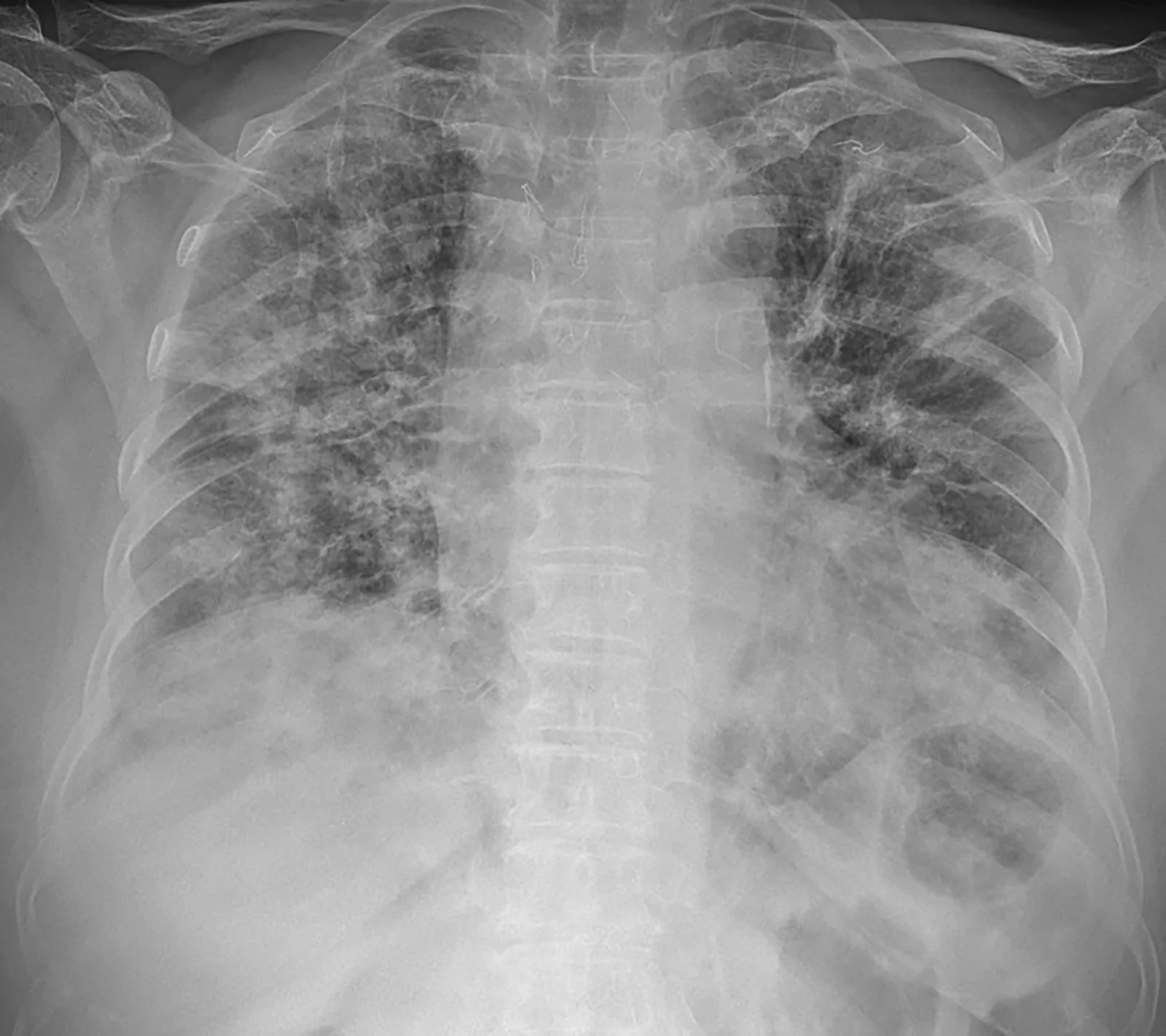
Initial chest X-ray of Case 2, showing diffuse bilateral infiltrates.

**Figure 7.**
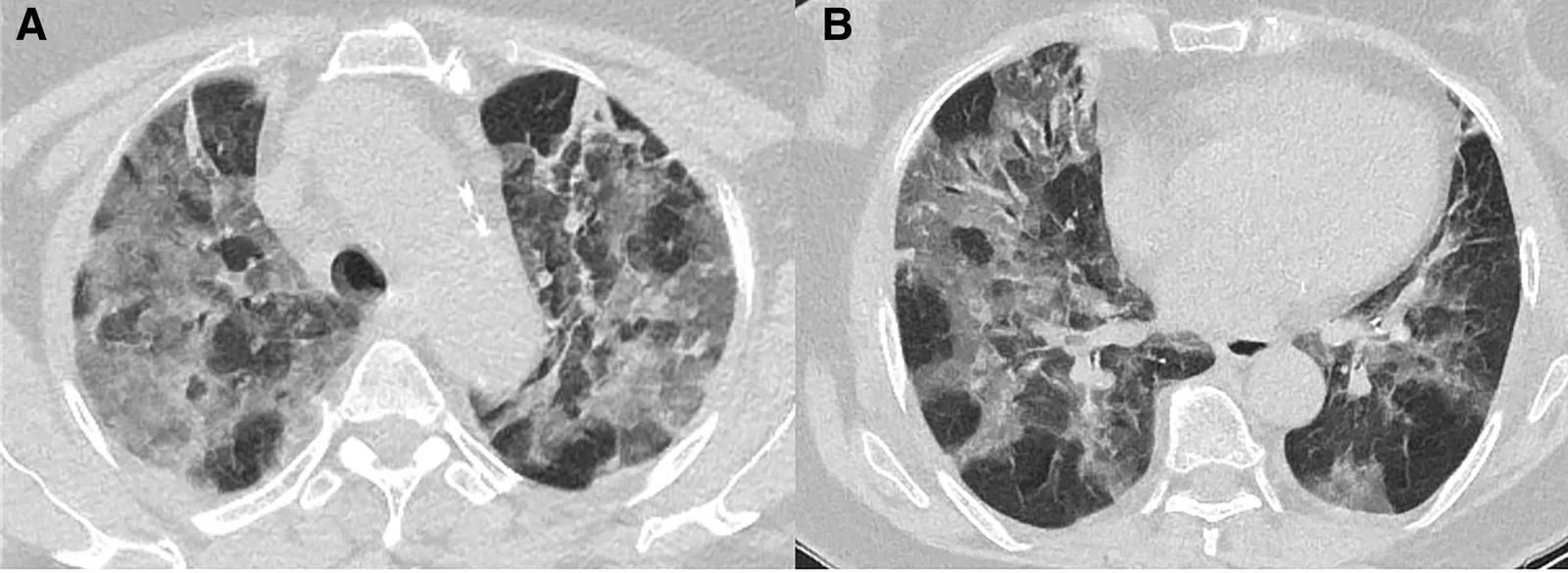
Initial chest computed tomography of Case 2, showing diffuse bilateral ground-glass opacities and consolidations in both lungs (A, B).

She received oxygen via high-flow nasal cannula for severe hypoxemia. Arterial blood gas analysis revealed a PaO_2_/FiO_2_ ratio of 140 mm Hg. Empirical piperacillin/tazobactam was initiated, and bronchoscopy was performed immediately thereafter for further diagnostic evaluation. BAL fluid revealed progressively bloodier aliquots, consistent with alveolar hemorrhage. Quantitative BAL fluid analysis showed a red blood cell count of 51,000 cells/µL and a total nucleated cell count of 610 cells/µL. Given the progressive hemoptysis and hypoxemia despite prior antibiotic therapy, together with bronchoscopic findings consistent with DAH, high-dose intravenous methylprednisolone (500 mg/day) was administered for 3 days, followed by tapering to 1 mg/kg/day. Then, methylprednisolone was further tapered according to hemoptysis resolution and oxygenation improvement. Following corticosteroid therapy, her hypoxemia improved rapidly, accompanied by improvement on chest radiograph (Fig. 8). Her hemoglobin level reached a nadir of 10.5 g/dL on hospital day 3, without requiring blood transfusion.

**Figure 8.**
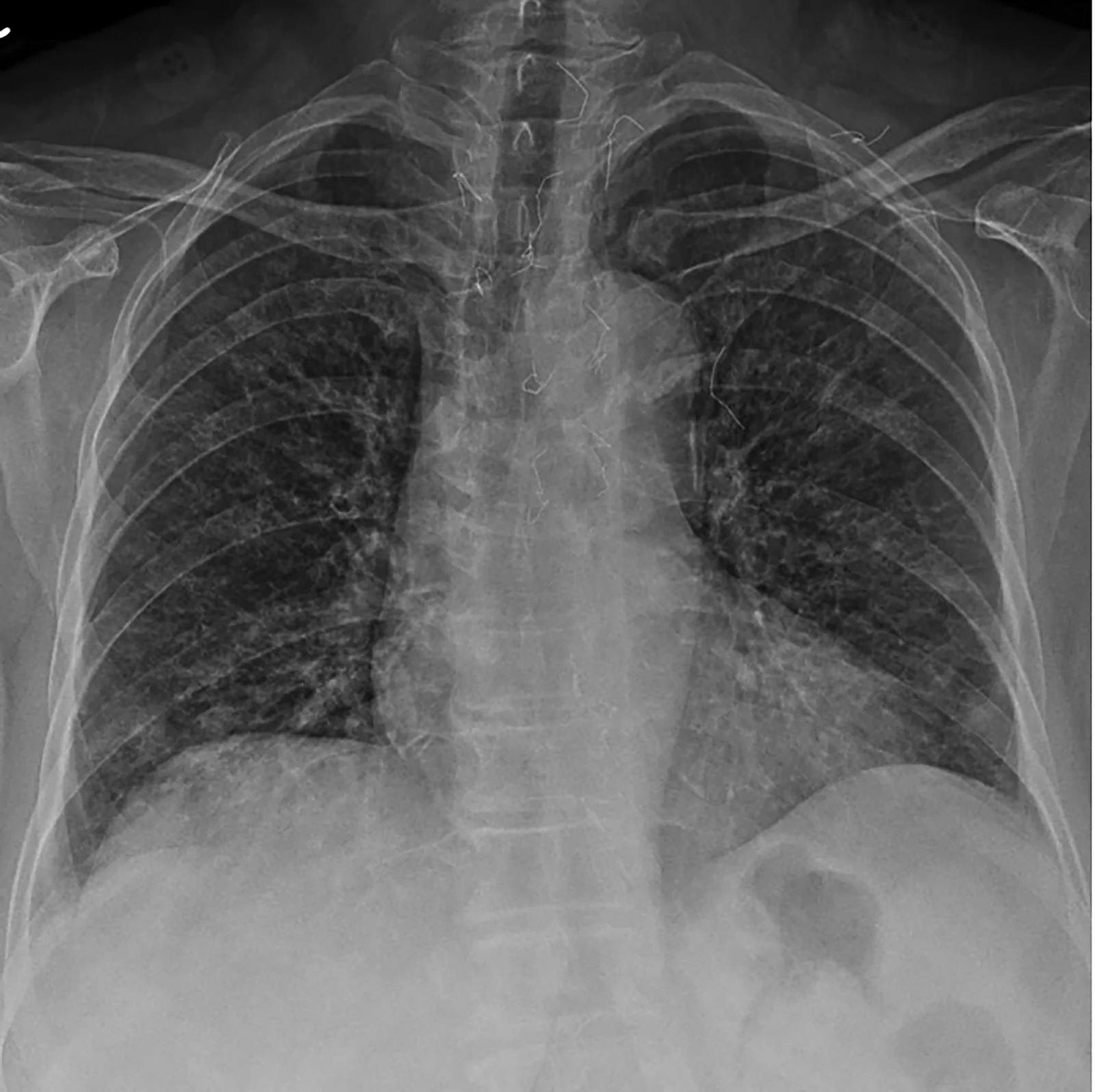
Chest radiograph of Case 2 on day 5 of corticosteroid therapy, demonstrating improvement of bilateral infiltrates with clinical recovery.

Microbiological studies of the BAL fluid were negative for other pathogens, although the effect of 5-day antibiotic exposure on bacterial culture yield could not be excluded. Multiplex respiratory viral PCR detected rhinovirus. Serologic tests for autoimmune markers, including anti-nuclear antibody and ANCA, were also negative. She was discharged on hospital day 8 on maintenance oral prednisolone 30 mg/day, which was tapered to 20 mg/day after 2 weeks and 10 mg/day after 6 weeks and discontinued approximately 3.5 months after discharge. No recurrent hemoptysis occurred during the 3 months following corticosteroid discontinuation. Figure 9 provides a timeline of the patient’s clinical course.

**Figure 9.**
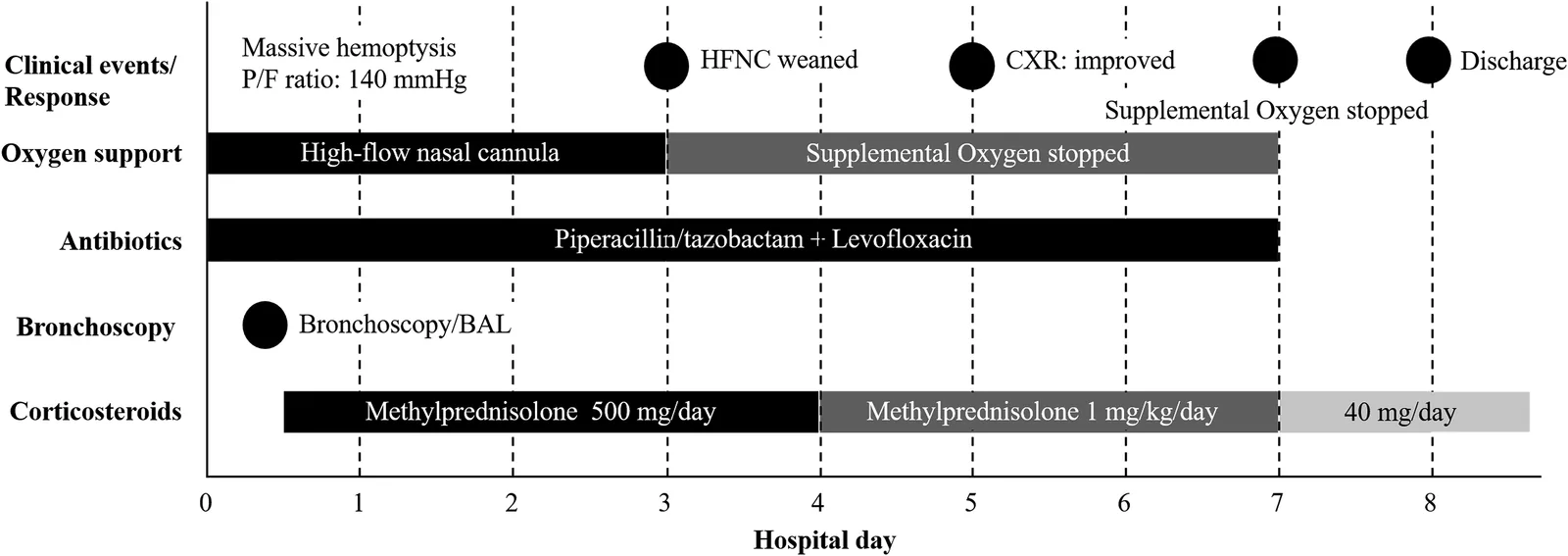
Timeline of the patient’s clinical course. BAL = bronchoalveolar lavage, CXR = chest X-ray, HFNC = high-flow nasal cannula, P/F ratio = the arterial partial pressure of oxygen to fraction of inspired oxygen ratio.

## 3. Discussion

We report 2 cases of life-threatening DAH in immunocompetent patients in whom rhinovirus was the sole pathogen detected in BAL fluid, without an alternative etiology. Both patients presented with massive hemoptysis and severe hypoxemia and showed marked clinical improvement after initiating corticosteroid therapy.

When DAH is suspected clinically, it is essential to confirm the diagnosis and underlying etiology. In this case report, DAH was initially suspected based on chest CT findings in both patients. Subsequent bronchoscopy with BAL revealed DAH with progressively hemorrhagic fluid. Microbiological testing of the BAL fluid identified only rhinovirus, with no evidence of connective tissue disease, malignancy, or other infections. Although the mechanism of infection-associated DAH remains unclear, several case reports have described it in immunocompetent hosts.^[5]^ Rhinovirus is generally considered a common pathogen of upper respiratory tract infections,^[6]^ but there is growing recognition of its potential to cause severe respiratory infections in immunocompetent patients.^[7–9]^ For example, Schmitz et al reported a case of DAH caused by acute *Mycoplasma pneumoniae* infection. Although a viral panel from the upper respiratory tract was positive for rhinovirus, *Mycoplasma pneumoniae* was considered the causative pathogen.^[10]^ Similarly, Shafi et al described DAH associated with marijuana smoking, in which the respiratory viral panel detected rhinovirus. The authors suggested that rhinovirus was not directly associated with DAH, but instead acted as a predisposing factor by causing airway inflammation that contributed to marijuana inhalation injury.^[11]^ Reina et al also reported DAH in an immunocompetent child with rhinovirus and human bocavirus coinfection, which was detected by pharyngeal swab PCR, making the independent contribution of rhinovirus uncertain.^[12]^ Unlike in previous reports, which considered rhinovirus an incidental finding or a predisposing factor, rhinovirus was the only pathogen detected in BAL from both patients in our cases, without evidence of an alternative etiology. BAL was performed according to standard guidelines, with the bronchoscope wedged in a segmental bronchus during sterile saline instillation and fluid retrieval to minimize contamination from proximal airway secretions.^[13]^ In contrast, BAL fluid positivity despite a negative nasal swab in Case 2 further supports lower respiratory tract involvement. Although causality cannot be established, these findings suggest a potential association between lower respiratory tract rhinovirus infection and DAH.

In cases of DAH with noninfectious etiology, corticosteroids and immunosuppressive agents are generally recommended^[14]^; however, evidence for their role in infection-associated DAH is limited. The use of corticosteroids in viral infections remains controversial, as they have been associated with prolonged viral shedding and an increased risk of secondary complications in patients with influenza pneumonia.^[15,16]^ By contrast, corticosteroids such as dexamethasone have improved survival in patients with COVID-19 requiring oxygen supplementation.^[17]^ Previous studies have also reported clinical improvements in pneumonia associated with rhinovirus infection.^[18,19]^ In the present report, both patients showed marked improvement in massive hemoptysis and severe hypoxemia following corticosteroid administration. Since the influence of concomitant interventions and the natural disease course cannot be fully excluded, and because the corticosteroid regimens differed between the 2 cases, a direct corticosteroid effect cannot be established. Nevertheless, the temporal association between corticosteroid therapy and clinical improvement suggests that corticosteroids might have contributed to patients’ recovery. Hence, further studies are warranted to clarify the role of corticosteroids in rhinovirus-associated DAH.

In conclusion, rhinovirus infection may be associated with life-threatening DAH accompanied by severe hypoxemia. In such cases, systemic corticosteroid therapy may contribute to clinical improvement, suggesting its potential therapeutic role.

## Author contributions

**Conceptualization:** Chang-Seok Yoon, Tae-Ok Kim, Hong-Joon Shin.

**Data curation:** Chang-Seok Yoon, Joon-Young Yoon, Bo-Gun Kho.

**Project administration:** Yong-Soo Kwon, Yu-Il Kim, Sung-Chul Lim, Hong-Joon Shin.

**Supervision:** Bo-Gun Kho, Tae-Ok Kim, Yong-Soo Kwon, Yu-Il Kim, Sung-Chul Lim.

**Writing – original draft:** Chang-Seok Yoon, Hong-Joon Shin.

**Writing – review & editing:** Chang-Seok Yoon, Joon-Young Yoon, Bo-Gun Kho, Tae-Ok Kim, Yong-Soo Kwon, Yu-Il Kim, Sung-Chul Lim, Hong-Joon Shin.
